# Effects of selenium source and level on the physiological response, reproductive performance, serum Se level and milk composition in gestating sows

**DOI:** 10.5713/ab.22.0104

**Published:** 2022-06-30

**Authors:** Xing Hao Jin, Cheon Soo Kim, Min Jin Gim, Yoo Yong Kim

**Affiliations:** 1 Department of Agricultural Biotechnology and Research Institute of Agriculture and Life Science, Seoul National University, Seoul 08826, Korea

**Keywords:** Litter Performance, Reproductive Performance, Selenium, Serum Selenium Level, Sows

## Abstract

**Objective:**

This study was conducted to evaluate the effects of selenium (Se) source and level on the physiological response, reproductive performance, serum Se level, and milk composition in gestating sows.

**Methods:**

A total of 54 multiparous sows (Yorkshire×Landrace) with average body weight (BW), backfat thickness (BF), and parity were assigned to one of five treatments with 10 or 11 sows per treatment using a 2×2 factorial arrangement with one additional treatment in a completely randomized design. Inorganic or organic Se (IS or OS) sources were added to the diet at 0.30 ppm and 0.50 ppm Se. A non-Se-fortified corn-soybean meal basal diet served as a negative control. Treatments were as follows: i) Control: corn-soybean based diet, ii) IS30: control+inorganic Se 0.30 ppm, iii) IS50: control+inorganic Se 0.50 ppm, iv) OS30: control+ organic Se ppm, and v) OS50: control+organic Se 0.50 ppm.

**Results:**

At day 21 of lactation, piglet weight and weight gain in the OS treatments were higher than those in the IS treatments (p<0.05). Meanwhile, adding 0.5 ppm Se also resulted in the same significant differences in piglet BW and weight gain (p<0.05). Colostrum and milk Se concentrations increased (p<0.05) with Se level for both Se sources but were greater when sows were fed organic Se (p<0.05). Except for 24 hours postpartum, the Se concentrations were higher when sows were fed organic Se (p<0.05). Sow serum Se content was greater as Se levels increased from 0.3 ppm to 0.5 ppm at day 110 of gestation, 24 hours postpartum and day 21 of lactation (p<0.05). The pig serum Se concentration increased as the dietary Se level increased (p<0.05) and was higher when the sow dietary Se source was organic (p<0.05). Organic Se 0.5 ppm also had the highest serum Se level at two measured points (p<0.05).

**Conclusion:**

Consequently, supplementation with organic Se or 0.5 ppm Se in a gestating diet could improve piglet performance, the Se status of sows and piglets and milk composition, but organic Se at 0.5 ppm is optimal.

## INTRODUCTION

Dietary selenium (Se) can improve antioxidant capacity, immune response, reproductive performance, and Se accumulation in the body [1]. The supplementation of organic or inorganic selenium in sow feed has attracted attention [2]. Compared with inorganic Se, organic Se has a higher absorption rate and bioavailability and lower toxicity [3]. It has been shown that supplementation with organic Se results in higher serum Se concentrations and greater Se accumulation both in sows and progeny [4,5]. Recently sow’s diet with organic Se has been widely discussed regarding swine diet [1].

Selenium deficiency can lead to a negative influence on the growth and reproduction of animals [1]. However, dietary Se up to 5 mg/kg in the diet may cause a toxic reaction [6,7]. NRC [8] has recommended dietary Se of gestating sows for 0.15 ppm in diet. However, FDA [9] suggested a swine diet of 0.3 ppm. Se retention and serum Se concentrations were increased in sows and their piglets as the dietary Se concentration increased from 0.1 ppm to 0.3 ppm [10,11]. However, the effect of Se supplementation at a high level (0.5 ppm) on sows and their offspring is unknown.

With dietary Se increased to a certain level, the serum Se concentrations of sows and piglets, colostrum and milk Se content were all increased, particularly the Se source was organic [12–14]. There are inconsistent reports on the interaction between Se source and level which were supplemented in gestation diet [15]. Moreover, late gestation and lactation or transition were considered in previous studies [16,17]. Chavez [18] reported continuous decline was observed in blood Se levels in gestating sows, and Se supplementation was necessary for gestating sows.

In the current study, the effect of Se source and level during whole gestation on sow reproductive performance, serum Se content of sows and piglets, and milk composition in sows were examined. We hypothesized that the long-term effect of maternal Se supplementation could improve the sow reproductive performance, litter performance and Se status of sows and piglets.

## MATERIALS AND METHODS

### Animals

All experimental procedures involving animals were conducted following the Animal Experimental Guidelines provided by the Seoul National University Institutional Animal Care and Use Committee (SNUIACUC; SNU-201112-3).

A total of 60 F1 multiparous sows (Yorkshire×Landrace) with an average body weight (BW) of 229.5 kg, an average backfat thickness (BF) of 22.2 mm, and an average parity of 4.8 were allotted to one of five treatments considering BW, BF, and pairty in a completely randomized design (CRD) after mating. All sows took two times of artificial insemination services according to the estrus cycle after weaning and checked their pregnancy at day 35 of gestation by ultrasound scanner (Donjin BLS, Gwangju-si, Gyeonggi-do, Korea). After pregnancy diagnosis, only 54 F1 multiparous sows were pregnant and continued to consume their treatment diets. During the experimental period, multiparous sows of third or over third parity were fed a 2.4 kg/d gestation diet.

### Experimental design and diet

The experiment was a 2×2 factorial arrangement with one additional treatment in a CRD. Inorganic (sodium selenite) or organic (Se-enriched yeast) Se sources were added to the diet at 0.30 or 0.50 ppm Se. A non-Se fortified corn-soybean meal basal diet served as a negative control. The experimental selenium sources added to the basal diet were sodium selenite (total Se 1,000 ppm, inorganic Se) and selenium yeast (total Se 1,000 ppm, organic Se, Sel-Plex; Alltech, South Brookings, SD, USA). Analyzed Selenium (Se) contents were as follows: i) Control: 0.07 ppm, ii) IS30: 0.41 ppm, iii) IS50: 0.65 ppm, iv) OS30: 0.44 ppm, and v) OS50: 0.63 ppm. All experimental gestation diets were formulated to contain 3,265 kcal of ME/kg, 12.00% crude protein, 0.74% total lysine, 0.23% total methionine, 0.75% calcium, and 0.60% total phosphorus. All other nutrients were formulated to meet or exceed the NRC [8] requirements. Table 1 shows the formulas and chemical compositions of gestation and lactation diet. All sows were fed the same commercial lactation diet during the lactation period.

### Animal management

All experimental sows (parity: 3 to 6) were fed an experimental diet once a day at 08:00 h and provided 2.4 kg per day during gestation and the gestation diet was decreased gradually 0.2 kg per day for 5 days before farrowing. After farrowing, sows were fed a lactation diet of 1, 2, 3, 4, and 5 kg/d as lactating age increased and fed a diet *ad libitum* until weaning.

All sows were accommodated in individual gestation stalls (2.20×0.64 m) where the indoor temperature was regulated to an average of 20°C by an automatic ventilation system. At day 110 of gestation, sows were moved from the gestation barn to farrowing crates (2.50×1.80 m) after washing and disinfecting their body, especially breast and vulva. None of the sows were treated with delivery inducer and they were assisted when dystocia occurred. The room temperature of the lactating barn was kept at 28°C±2°C and the baby house under heating lamp was kept at 32°C±2°C. The air condition of the lactating barn was regulated automatically by the ventilation system and air-conditioner. After weaning, the sows were moved to a breeding barn for the next oestrus cycle.

After farrowing, piglets were cross-fostered within treatment until 24 h postpartum to balance the suckling intensity of sows with equalization of litter size, and thus to minimize any effect of initial litter size potentially affecting litter growth. Cutting of the umbilical cord and tail and castration were conducted 3 days after birth, and piglets were injected with 150 ppm Fe-dextran (Gleptosil; Alstoe, York, UK) injection. All piglets were not fed creep feed during the whole lactation period. Weaning was performed at approximately 24±3 d.

### Performance of sow

The BW and BF of sows were measure at day 70, 90, and 110 of gestation, 24 h postpartum, and day 21 of lactation. The BW of the sow was measured by electric scale (CAS Co. Ltd., Yangju-si, Gyeonggi-do, Korea) for sow and BF was measured at the P_2_ position (mean value form both side of the last rib and 65 mm away from the backbone) by an ultrasound device (Lean Meter; Renco Corp., Golden Valley, MN, USA). Daily feed wastage was recorded during lactation and lactation feed intake was measured when measuring the BW and BF of lactating sows at day 21 of lactation. The weaning to estrus interval (WEI) of sows, as an important parameter for evaluating reproductive performance, was measured after weaning.

### Reproductive performance

After farrowing, the number of piglets for total born, stillbirth, mummy, alive piglets was recorded, and the BW of alive piglets, stillborn, and mummy was measured by electric scale (CAS Co. Ltd., Korea). When measuring the BW of piglets, ear notching was practiced for the experiment.

### Litter performance

After ear notching, cross-fostering of the piglets within the same treatment was performed until 12 h postpartum for equalizing litter size. The number and BW of piglets was measured again at day 21 of lactation to calculate litter weight, piglet weight and both weight gain.

### Blood samples

Blood collection from sows based on similar BW and BF (n = 4 for each treatment) was taken by venipuncture of the jugular vein using 10 mL disposable syringes at day 70, 90, 110 of gestation, 24 h postpartum and day 21 of lactation. Blood from suckling piglets (n = 16 for each treatment) was collected from the anterior vena cava using 3 mL disposable syringes at 24 h postpartum and 5 mL disposable syringes at day 21 of lactation. All blood samples were enclosed in serum tubes (SSTTMII Advance; BD Vacutainer, Becton Dickinson, Plymouth, UK) as well as ethylenediaminetetraacetic acid tube (BD Vacutainer K2E; Becton Dickinson, UK) and centrifuged at 3,000 rpm and 4°C for 15 min (5810R; Eppendorf, Hamburg, Germany) after clotting at room temperature for 30 min. The upper liquid (serum) of the blood was separated to a microtube (Axygen, Union City, CA, USA) and stored at −20°C freezer until later analysis.

### Milk composition

Colostrum and milk samples were taken from functional mammary glands of each sow at 24 hours post-farrowing and day 21 of lactation, respectively. Injection of 5 mL of oxytocin (Komi oxytocin inj.; Komipharm International Co., Ltd., Siheung-si, Gyeonggi-do, Korea) was into the blood vessels of the sow’s ear. Colostrum and milk were collected in 50 mL conical tubes (SPL Life Sciences Co., Ltd., Pocheon-si, Gyeonggi-do, Korea) from the first and second teats. After collection, samples were stored in a freezer (−20°C) until further analysis. Proximate analysis of colostrum and milk was conducted using Milkoscan FT120 (FOSS Electric, Hillerød, Denmark).

### Se analytical methods

Colostrum, milk, serum and diets were conducted according to the fluorometric method outlined by AOAC [19]. After the wet ashing of samples with nitric acid and perchloric acid at 160 degrees °C for about 2.5 to 3 hours and the reduction with 6 mol/L hydrochloric acid, selenium was determined by 2,3-diamino-naphthalene fluorescence reaction.

### Statistical analysis

All of collected data were carried out by least squares mean comparisons and were evaluated with the general linear model (GLM) procedure of SAS [20]. Individual sows, whole litter weight, and average pig weight within litter were considered the experimental units. All collected data were analyzed by a one-way analysis of variance. Contrasts were used to compare control to other treatments. The four treatments that had organic or inorganic Se at the 0.30 and 0.50 ppm Se were analyzed as a 2×2 factorial, with the main effects and interactions evaluated. Differences among means were declared significant at p<0.05 and highly significant at p<0.01 and the determination of tendency for all analyses was p≥0.05 and p<0.10.

## RESULTS

### Performance of sow

The effects of selenium source and level on performance in gestating sows were shown in Table 2. Body weight, BF, lactation feed intake and WEI of sows were not affected by dietary Se source or Se level during gestation and lactation periods. None of the treatments resulted in significant differences in the performance of sows.

### Reproductive performance

Sow reproductive performance in response to Se source and level is described in Table 3. The total number of pigs born, the number of stillbirths and the number of born alive were unaffected by dietary treatment. Litter birth weight and individual birth weights also showed no significant differences among all treatments.

### Litter performance

The effects of Se sources and levels on the litter performance of sows are shown in Table 4. At day 21 of lactation, piglet weight and weight gain in the organic source were higher than those in the inorganic treatments (p<0.05; p<0.05; respectively). Meanwhile, adding high level of Se 0.50 ppm also was higher than adding a low level of Se 0.3 ppm in piglet BW and weight gain at day 21 of lactation (p<0.05; p<0.05; respectively).

### Milk composition

The effects of dietary Se sources and levels on the chemical composition of colostrum and milk in sows are shown in Table 5. As a result of the present study, Se content in colostrum was increased by organic Se or high levels of Se (p< 0.05; p<0.05, respectively) which resulted in a significant Se source×Se level interaction (p<0.05). At day 21 of lactation, the milk Se concentration in the organic Se treatment was higher than that in the inorganic Se treatment (p<0.05) and an increase of Se content in milk was observed as the amount of Se level increased (p<0.05). Among all treatments, organic Se 0.5 ppm also had the highest serum Se content in colostrum and milk during the lactating period (p<0.05; p<0.05, respectively).

### Serum selenium concentration in gestating sows

The effects of selenium source and level on serum Se concentration in sows during the gestation-lactation period are shown in Table 6. The Se concentrations at days 70, 90, and 110 of gestation and day 21 of lactation were higher when sows were fed organic Se instead of inorganic Se (p<0.05; p<0.05; p<0.05; p<0.05; respectively). According to Se level, the Se content was greater as Se level increased from 0.3 ppm to 0.5 ppm at day 110 of gestation, 24 hours postpartum and day 21 of lactation (p<0.05; p<0.05; p<0.05; respectively). In addition, the interaction between the Se source and the level was observed at day 21 of lactation (p<0.05). Except for day 90 of gestation, the organic Se 0.5 ppm treatment had a higher serum Se level than the other treatments (p<0.05).

### Serum selenium concentration in piglets

The effects of selenium source and level on serum Se concentration in piglets are shown in Table 7. During the suckling period, the pig serum Se concentration increased as the dietary Se level increased (p<0.05; p<0.05; respectively) and was higher when the sow dietary Se source was organic (p< 0.05; p<0.05). This resulted in an interaction response at 24 hours postpartum(p<0.05) but not at day 21 of age. Among all the treatments, organic Se 0.5 ppm also had the highest serum Se level at two measured points (p<0.05; p<0.05, respectively).

## DISCUSSION

Many studies have been conducted to evaluate the effects of different Se sources nor levels on the performance of sows [4,5,11,13]. Dietary Se source fed to gestating sows did not affect BW or BF thickness during the gestation and lactation periods [21,22]. As the dietary Se level (0 ppm, 0.15 ppm, 0.30 ppm Se) increased during late gestation and lactation, BW and BF showed no significant differences after farrowing and 14 days of lactation [13].

Moreover, Mahan and Peter [11] also reported that BW and average daily feed intake were not affected by Se source or Se level when organic Se (0.15 or 0.3 ppm) and inorganic Se (0.15 or 0.3 ppm) were provided in gestating diets. In agreement with the above, the present study indicated that maternal Se source and level did not influence the performance of sows during the whole experimental period. In addition, control and other treatments were similar in performance of multiparous sows (over 3 parties) and demonstrated that no addition of dietary Se in gestating diet had no detectable effects on sow’s body condition within one reproducitve cycle.

Falk et al [17] reported that feed intake in gestating sows fed with organic Se was higher than that in sows fed with inorganic Se and was not influenced by different Se levels at the same Se source during the lactation period. In this study, feed intake could not be observed in organic Se treatment even if Se yeast were provided.

Feed intake has strong correlations with an increase in BW in the gestation period and loss of BW after farrowing [23]. In the present study, changes in BW and BF showed no significant differences during the whole experimental period and remained within normal ranges for sow maintenance and milk production. Maximum Se level of this study was 0.5 ppm and could not lead to low feed intake due to toxic problems. During lactation, low feed intake could mobilize more body reserves to maintain milk production, resulting in catabolic state [23] and an increase of WEI in subsequent reproduction cycles [24,25]. It was also known that WEI was affected by BW loss and litter size during lactation [26]. In this experiment, there were no significant differences in WEI among treatments.

In current study, reproductive performance was not affected by Se source and level in throughout the entire gestation period, as with the report by Svoboda et al [27], who found no difference in sow reproductive performance when sows were fed inorganic Se or Se yeast. Yoon and McMillan [28] also found that Se source at 0.3 ppm did not affect litter size or piglet birth weight when experimental diets were provided from day 60 of prepartum until weaning at 14 days. However, sows fed the organic Se diet had greater litter birth weights than sows fed the inorganic Se diet during whole gestation [14]. An improvement in the reproductive performance of sows could be explained by the results of Fortier et al [29], who demonstrated that supplementation with organic Se in the diets of gilt from estrus to day 30 of gestation could increase embryonic length and weight. In the present study, multiparous sows (3 or over 3 party) were used and these variations were probably due to sow parity, experimental period, and Se sources including inorganic and organic forms.

Zhan et al [16] and Hu et al [30] reported that weaned BW and weight gain were improved when sows were fed organic Se 0.3 ppm compared with inorganic Se 0.3 ppm during late gestation and lactation. A recent study by Falk et al [31] also showed that BW of piglets was improved by organic Se and high level of Se. The results of the present study were in an agreement with previous studies, and this study indicated that organic Se or a high level of Se could affect positively piglet BW and weight gain at day 21 of age. Nutrient fortified feed will improve the composition of both colostrum and milk subsequently leading to a better performance of their progeny [32]. Falk et al [17] also reported that the enhancement of growth performance and survival rate of piglets were found as Se content in colostrum and milk increased. Therefore, an improvement in piglet performance must be in relation to an increase of Se content in colostrum and milk which was consistent with the results regarding milk composition shown in Table 5.

Milk composition is more affected by nutrients consumed in the gestating period [33]. When organic Se was fed to gestating sows colostrum and milk had higher Se levels [11,28]. More of the Se absorbed from the organic Se source was effectively incorporated into milk [34]. This mechanism explains the greater increase in colostrum and milk Se contents when the organic source was provided in the present study. The current NRC [8] standards for Se are 0.15 ppm in the diet for reproducing sows, but the supplemental level frequently used in the feed industry is 0.3 ppm. Our data showed that there were greater increases in colostrum and milk Se contents as Se levels increased up to 0.5 ppm. In addition, among all treatments, organic Se 0.5 ppm had a highest serum Se content in colostrum and milk during lactating period. It also strongly verified another theory that organic Se sources with increasing Se levels could be more effectively deposited during gestation and more easily transferred from mammal gland to milk. Moreover, sows fed inorganic Se 0.3 ppm had milk Se concentrations similar to those of sows fed inorganic Se 0.5 ppm and both lower than organic Se concentrations at 21 days of lactation, suggesting that a higher dietary level when using inorganic Se would not produce much of an increase in milk Se content.

Svoboda et al [27] and Hu et al [30] found that Se content in plasma and serum of sows was higher when sows were fed organic Se compared with sows fed inorganic Se in the gestating diet. Kim and Mahan [5] reported that when gilts were fed different Se levels (0, 3, 7, 10 ppm), serum Se levels was linearly increased as Se levels increased. Mahan and Kim [35] and Mahan and Peter [11] also reported that serum Se levels were consistently higher as Se levels increased and when organic Se was fed compared with inorganic Se. Based on the above, the changes in serum Se in the present study were similar to those in previous studies. In the present study, organic Se 0.5 ppm treatment had the highest serum Se level at any measured period. There are considerable commonalities in serum Se levels between inorganic Se 0.3 ppm and inorganic Se 0.5 ppm at 24 hours postpartum and day 21 of lactation. These data indicated that an increase of inorganic Se level could not improve sow serum Se content during the lactation period which was contradiction to organic Se level. Moreover, lower serum Se content was observed at day 90 of lactation in this study compared with other measured points. From day 90 of gestation, sows had significant physiological changes in the mammary gland development, preparation for colostrum secretion, and rapid growth of the fetus and then more nutrients could be mobilized to the gland or fetus [36]. That was why a lower numerical value in the serum Se level was observed at day 90 of gestation.

Newborn piglets mainly absorbed nutrients through colostrum and milk [37], and sows fed different Se sources and levels could improve the selenium status of piglets [38]. In this study, the results are generally consistent with previous reports by Quesnel et al [12] and Mahan and Peter [11], who found that organic Se treatments had higher piglet Se concentrations than inorganic Se when diets were supplied from gestation and lactation. Mahan [13] also reported that the Se content of piglets in serum at birth and weaning age could be increased linearly as the Se level increased. In the present study, control had a lower serum Se concentration than the other Se supplementation treatments. The pig serum Se concentrations in the treatments of sows fed inorganic Se at the two levels were similar at 24 hours postpartum, indicating that sows were fed inorganic Se could not enhance the Se status of piglets. However, at day 21 of age, IS 0.5 treatment had a higher Se concentration than IS 0.3 treatment which may be caused by increasing milk consumption as piglet BW increased or milk Se concentration.

## CONCLUSION

The supplementation of organic Se or 0.5 ppm Se in a gestating diet could improve piglet performance, milk composition and the Se status of sows and piglets, but organic Se at 0.5 ppm in particular was more effective. Further research should be focused on the influence of combined Se sources and levels on reproductive performance, piglet performance and the Se status of sows and piglets.

## Figures and Tables

**Table 1:** Formula and chemical compositions of gestation diet^1)^ and lactation diet

Items	Gestation diet^2)^	Lactation diet
Ingredients (%)		
Corn	75.61	65.35
Soybean meal, 48% CP	13.23	27.23
Wheat bran	5.51	2.01
Tallow	1.75	2.42
L-lysine HCl (50%)	0.37	-
DL-methionine (99%)	0.03	-
Dicalcium phosphate	1.58	1.26
Limestone	1.22	1.13
Vit. Mix^3)^	0.10	0.10
Min.Mix^4)^	0.10	0.10
Choline chloride-50%	0.10	0.10
Salt	0.40	0.30
Sum	100.00	100.00
Chemical composition^5)^		
ME (kcal/kg)	3,265	3,300.06
Crude protein (%)	12.00	17.20
Lysine (%)	0.74	0.94
Methionine (%)	0.23	0.28
Ca (%)	0.75	0.75
Total P (%)	0.60	0.60

CP, crude protein; ME, metabolizable energy.

1) Sodium selenite and Se yeast contained total selenium 1,000 ppm respectively and were added to basal diet to achieve the appropriate treatment levels.

2) Formulated to % Lys, %, Met, % Ca and % P (total).

3) Provided the following per kilogram of gestation diet: vitamin A, 8,000 IU; vitamin D_3_, 1,600 IU; vitamin E, 32 IU; d-biotin, 64 g; riboflavin, 3.2 mg; calcium pantothenic acid, 8 mg; niacin, 16 mg; vitamin B_12_, 12 g; vitamin K, 2.4 mg. Provided per kg of lactation diet: vitamin A, 10,000 IU; vitamin D_3_, 1,900 IU; vitamin E, 80 IU; vitamin K_3_, 3.25 mg; thiamine (vitamin B_1_), 2.00 mg; riboflavin (vitamin B_2_), 7.0 mg; pantothenic acid (vitamin B_5_), 27.5 mg; niacin (vitamin B_3_), 36 mg; pyridoxine (vitamin B_6_), 3.75 mg; d-biotin, 0.35 mg; folic acid, 2.25 mg; vitamin B_12_, 0.03 mg.

4) Provided the following per kilogram of gestation diet: I, 0.3 mg; Mn, 24.8 mg; CuSO4, 54.1 mg; Fe, 127.3 mg; Zn, 84.7 mg; Co, 0.3 mg. Provided per kg of lactation diet: Se, 0.15 mg; I, 0.3 mg; Mn, 37 mg; Cu, 11 mg; Fe, 150 mg; Zn, 85 mg; Co, 2 mg.

5) Calculated values.

**Table 2:** Effects of selenium source and dietary level in gestation diet on body weight, backfat thickness, weaning to estrus interval and lactation feed intake of sows^1)^

Items	Control	Inorganic Se (ppm)		Organic Se (ppm)		SEM	Diet	p-value		
		
		0.3	0.5	0.3	0.5			Source (S)	Level (L)	S×L
No. of sow bred	12	12	12	12	12					
No. of sows farrowed	10	11	11	11	11					
Body weight (kg)										
At mating	235.00	234.83	233.33	233.40	234.83	4.36	1.00	0.99	0.99	0.89
35 d	236.33	238.33	236.83	237.17	238.17	5.09	1.00	0.99	0.98	0.92
110 d	273.53	272.83	273.73	276.00	278.00	4.61	1.00	0.74	0.90	0.96
Change (0 to 110 d)	38.53	38.00	40.40	42.60	43.17	2.00	0.99	0.69	0.94	0.92
24 h postpartum	252.36	249.43	251.23	253.47	256.40	5.96	1.00	0.75	0.87	0.97
21 d of lactation	245.98	245.33	243.80	244.83	246.80	6.28	1.00	0.93	0.99	0.91
Change (0 to 21 d)	−6.83	−4.10	−7.43	−8.63	−9.60	1.05	0.87	0.13	0.31	0.57
Backfat thickness (mm)										
At mating	19.67	19.83	20.03	19.89	19.69	0.28	1.00	0.83	1.00	0.77
35 d	20.33	21.00	20.83	20.83	23.17	0.70	0.94	0.49	0.49	0.43
110 d	23.70	23.87	24.00	24.39	25.53	0.87	0.98	0.62	0.76	0.81
BF gain (0 to 110 d)	4.03	4.04	3.97	4.50	5.85	0.82	0.95	0.54	0.74	0.71
24 h postpartum	21.95	22.50	23.08	23.61	23.71	0.74	0.96	0.62	0.84	0.89
21 d of lactation	19.72	20.72	20.37	20.10	19.46	0.43	0.96	0.44	0.61	0.88
BF change (0 to 21 d)	−2.23	−1.78	−2.71	−3.51	−4.25	0.55	0.48	0.18	0.47	0.93
Lactation feed intake (kg/d)	5.95	5.86	5.92	6.03	6.04	0.32	1.00	0.85	0.96	0.97
WEI (d)	4.87	4.79	5.07	4.87	4.97	0.11	0.25	0.26	0.34	0.42

SEM, standard error of mean; BF, backfat thickness; WEI, weaning to estrus interval.

1) A total of 60 multiparous sows were bred, but only 54 sows were successful of pregnancy.

**Table 3:** Effects of selenium source and dietary level in gestation diet on reproductive performance of sows

Items	Control	Inorganic Se (ppm)		Organic Se (ppm)		SEM	Diet	p-value		
		
		0.3	0.5	0.3	0.5			Source (S)	Level (L)	S×L
No. of sows	10	11	11	11	11					
No. of piglets										
Total born	13.0	13.0	13.3	12.3	12.7	0.32	0.86	0.37	0.65	1.00
Stillbirth	0.6	0.5	0.4	0.5	0.4	0.15	0.65	0.63	0.61	0.63
Mummy	0.4	0.2	0.3	0.2	0.3	0.12	0.56	0.75	0.36	0.58
Born alive	12.0	12.3	12.3	11.6	12.0	0.25	0.81	0.24	1.00	1.00
Litter weight										
Total litter weight (kg)	18.43	17.04	19.03	16.60	17.74	0.70	0.71	0.58	0.33	0.78
Alive litter weight (kg)	17.29	16.33	18.18	16.00	17.05	0.63	0.75	0.61	0.31	0.77
Piglet weight										
Piglet birth weight (kg)	1.44	1.32	1.47	1.37	1.46	0.04	0.47	0.83	0.13	0.67

SEM, standard error of mean.

**Table 4:** Effects of selenium source and dietary level in gestation diet on litter performance

Items	Control	Inorganic Se (ppm)		Organic Se (ppm)		SEM	Diet	p-value		
		
		0.3	0.5	0.3	0.5			Source (S)	Level (L)	S×L
No. of piglets										
After cross-foster^1)^	12.0	12.3	12.0	12.0	12.0	0.15	0.96	0.63	0.64	0.58
21 day of lactation	10.6	10.6	10.3	10.7	10.3	0.23	0.97	1.00	0.54	1.00
Litter weight (kg)										
After cross-foster	17.29	16.33	17.69	16.87	17.47	0.48	0.80	0.88	0.38	0.73
21 days of lactation	56.45	55.79	58.05	59.52	60.70	1.46	0.87	0.35	0.60	0.87
Litter weight gain (0 to 21 d)	39.16	39.47	40.36	42.65	43.27	1.24	0.86	0.30	0.79	0.96
Piglet weight (kg)										
After cross-foster	1.44	1.32	1.47	1.41	1.46	0.04	0.53	0.64	0.20	0.49
21 day of lactation	5.29	5.23	5.62	5.58	5.87	0.08	0.29	0.01	0.01	0.57
Piglet weight gain (0 to 21 d)	3.85	3.91	4.14	4.17	4.41	0.07	0.54	0.03	0.05	0.93

SEM, standard error of mean.

1) After cross-fostering day within 24 hours postpartum.

**Table 5:** Effects of selenium source and dietary level in gestation diet on chemical compositions on colostrum and milk of sows

Items	Control	Inoganic Se (ppm)		Organic Se (ppm)		SEM	Diet	p-value		
		
		0.3	0.5	0.3	0.5			Source (S)	Level (L)	S×L
Selenium (ppm)										
Colostrum	0.051^a^	0.071^b^	0.099^c^	0.136^d^	0.200^e^	0.015	0.001	0.001	0.001	0.018
Milk (21 d)	0.026^a^	0.043^ab^	0.052^ab^	0.079^b^	0.106^c^	0.010	0.022	0.001	0.026	0.227
Casein (%)										
Colostrum	7.75	7.78	7.73	7.76	8.12	0.19	0.96	0.68	0.73	0.65
Milk (21 d)	3.94	3.93	4.02	4.01	4.01	0.07	0.99	0.83	0.77	0.75
Fat (%)										
Colostrum	5.64	5.65	5.64	5.62	5.72	0.25	1.00	0.97	0.94	0.92
Milk (21 d)	7.05	6.42	7.53	7.01	6.94	0.28	0.70	1.00	0.40	0.34
Protein (%)										
Colostrum	11.88	11.86	11.89	11.86	12.04	0.14	0.99	0.83	0.75	0.83
Milk (21 d)	4.86	4.77	4.92	4.86	4.86	0.08	0.99	0.93	0.70	0.71
Lactose (%)										
Colostrum	3.51	3.50	3.51	3.47	3.42	0.04	0.95	0.53	0.83	0.75
Milk (21 d)	5.62	5.78	5.60	5.70	5.79	0.04	0.51	0.44	0.55	0.09
Total solid (%)										
Colostrum	23.38	23.37	23.38	23.40	23.99	0.27	0.94	0.62	0.64	0.65
Milk (21 d)	19.11	18.49	19.89	19.12	19.11	0.35	0.75	0.92	0.37	0.37
Solid not fat (%)										
Colostrum	17.01	17.06	17.05	17.10	17.10	0.32	1.00	0.95	1.00	0.99
Milk (21 d)	11.13	11.22	11.20	11.23	11.37	0.08	0.94	0.61	0.73	0.65

SEM, standard error of mean.

a-e Means with different superscripts in the same row significantly differ (p<0.05).

**Table 6:** Effects of selenium source and dietary level in gestation diet on serum Se concentration of gestating and lactating sows

Items	Control	Inorganic Se		Organic Se		SEM	Diet	p-value		
		
		0.3	0.5	0.3	0.5			Source (S)	Level (L)	S×L
Serum selenium (ppm)										
At mating	------------------------------------------ 0.159 -------------------------------------									
70 d	0.144^a^	0.157^ab^	0.175^ab^	0.223^b^	0.229^c^	0.010	0.001	0.001	0.126	0.474
90 d	0.081	0.087	0.099	0.106	0.138	0.011	0.076	0.045	0.119	0.449
110 d	0.174^a^	0.199^ab^	0.225^b^	0.234^bc^	0.276^c^	0.012	0.007	0.034	0.013	0.597
24 h postpartum	0.146^a^	0.164^ab^	0.162^ab^	0.197^c^	0.247^d^	0.010	0.001	0.100	0.002	0.070
21 d of lactation	0.177^a^	0.230^ab^	0.257^ab^	0.304^b^	0.309^b^	0.018	0.001	0.002	0.002	0.001

SEM, standard error of mean.

a-d Means with different superscripts in the same row significantly differ (p<0.05).

**Table 7:** Effects of selenium source and dietary level in gestation diet on serum Se concentration of piglets

Items	Control	Inorganic Se (ppm)		Organic Se (ppm)		SEM	Diet	p-value		
	
		0.3	0.5	0.3	0.5			Source (S)	Level (L)	S×L
Serum selenium (ppm)										
24 h postpartum	0.025^a^	0.039^ab^	0.044^ab^	0.075^b^	0.115^c^	0.010	0.001	0.002	0.003	0.019
21 d of lactation	0.014^a^	0.037^ab^	0.063^b^	0.124^c^	0.142^d^	0.018	0.033	0.001	0.023	0.409

SEM, standard error of mean.

a-d Means with different superscripts in the same row significantly differ (p<0.05).
